# Poor correlation between alcohol concentration in oral fluid and breath in subjects consuming beverages immediately before testing

**DOI:** 10.11613/BM.2022.010902

**Published:** 2021-12-15

**Authors:** Hallvard Gjerde, Anne Line Bretteville-Jensen, Håvard Furuhaugen

**Affiliations:** 1 Section for Drug Abuse Research, Department of Forensic Sciences, Oslo University Hospital, Oslo, Norway; 2 Department of Alcohol, Tobacco and Drugs, Norwegian Institute of Public Health, Oslo, Norway

**Keywords:** breath tests, ethanol, feasibility studies, saliva

## Abstract

**Introduction:**

In previous research projects and clinical settings, alcohol analysis in oral fluid (saliva) has been used as an alternative to breath or blood alcohol testing. In this study we examined whether it is possible to obtain clinically relevant data regarding alcohol consumption in individuals who recently consumed alcohol by analysing oral fluid samples when the recommended rinsing of the mouth is impossible before sample collection.

**Materials and methods:**

We conducted a study of 89 nightclub patrons in Norway. Before collecting oral fluid samples and performing breath alcohol testing, participants were required to drink a glass of water to remove residual alcohol from the mouth. Oral fluid samples were collected with the Quantisal oral fluid collection device and analysed using an enzymatic method for alcohol. The alcohol concentration in the neat (undiluted) oral fluid was then calculated. Breath alcohol testing was performed using Lion Alcolmeter 500 instruments.

**Results:**

No false-negative or false-positive results for alcohol were detected in the oral fluid when compared with those in the breath. The Intraclass Correlation Coefficient of 0.40 indicated a poor correlation between alcohol concentrations in the two sample types.

**Conclusions:**

The procedure for collecting oral fluid was suitable for the qualitative determination of alcohol intake but not for quantitative assessment. We recommend that oral fluid samples should not be used for estimating blood or breath alcohol concentrations in people who have recently consumed alcohol or non-alcoholic beverages, as recommended in the instructions for use.

## Introduction

Breath alcohol testing is commonly performed to estimate the blood alcohol concentration (BAC), as it is less invasive, affords rapid results, and has shown a good correlation with the actual alcohol concentrations in blood samples (*1*). Accordingly, this method is commonly employed by law enforcement officers, either for initial screening followed by the collection of blood samples for accurate BAC determination or as evidential breath testing using instruments with good accuracy and specificity approved for the specific purpose.

In some research projects, as well as in clinical settings, alcohol analysis in oral fluid (saliva) has been employed as an alternative to breath or blood alcohol testing for practical reasons, given the good correlation between alcohol concentrations in oral fluid and blood (*2*, *3*). In addition, point-of-care devices for alcohol in oral fluid are available, for example, the Q.E.D. device from Orasure Technologies, Inc. (Bethlehem, PA, USA) (*4*). Therefore, alcohol testing of oral fluid samples has been used to detect alcohol use among trauma patients and in roadside surveys on alcohol use among drivers in road traffic (*5*, *6*).

Among subjects who recently consumed alcohol, an accurate estimation of BAC based on oral fluid or breath testing remains challenging. The breath alcohol concentration reflects the alcohol concentration in the pulmonary and arterial blood, but the measured concentration may be falsely elevated if there is residual alcoholic beverage present in the mouth; therefore, breath alcohol testing should be performed at least 20 min after the last sip of the alcoholic drink (*7*). This is often not feasible in clinical or research settings. When collecting oral fluid, any residual alcohol in the mouth is included in the sample. Rinsing the mouth with water before performing breath alcohol testing will reduce the alcohol content in the mouth and give a breath alcohol concentration that more accurately reflects the BAC. However, this may be practically difficult for some trauma patients and participants in certain research settings. One challenge with estimating alcohol in oral fluid after rinsing the mouth is that it may take at least 15 min to eliminate residual water and re-establish the equilibrium between oral fluid and blood (*8*).

The instrument manufacturers recommend that oral fluid samples should be collected at least 10 minutes after consumption of food and beverage and analysis of breath alcohol should be done at least 20 min after drinking or taking anything by mouth (*9*, *10*). Our hypothesis was that clinically relevant information regarding alcohol consumption can still be obtained by analysing oral fluid samples even in situations where the patient or study participant had recently consumed alcohol and it is practically impossible to rinse the mouth with water and wait 15 min prior to oral fluid collection. To test this hypothesis, we compared the alcohol concentration in breath and oral fluid in a situation with heavy alcohol consumption, namely among nightclub visitors.

## Materials and methods

### Subjects

The study was performed to determine the diagnostic accuracy of alcohol use in oral fluid samples when compared with breath testing. A convenience sample of 95 subjects who gave informed consent was recruited at nightclubs in Norway on Friday and Saturday nights during the summer of 2017. The study was approved by the Regional Committee for Medical and Health Research Ethics (approval no. 2016/337).

### Methods

Trained members of the research team performed the collection of oral fluid and breath alcohol testing. Before collecting oral fluid and breath alcohol testing, the participants were asked to drink a glass of water to rinse their mouths.

Breath alcohol testing was performed using Lion Alcolmeter 500 instruments (Lion Laboratories Limited, Vale of Glamorgan, United Kingdom). This breathalyser type uses a fuel cell sensor to determine the alcohol concentration. The instrument was programmed to provide a readout of the estimated BAC (g/L) using a blood-to-breath ratio of 2000:1. Next, we back-calculated the measured alcohol concentrations in the breath (mg/L). An alcohol concentration exceeding 0.05 mg/L exhaled breath was regarded as positive. The instruments were calibrated and controlled by the manufacturer shortly before the study. Published validation data indicate good precision (difference between replicates < 0.1 g/L) and good correlation with alcohol concentrations in blood (r = 0.97) (*11*).

Oral fluid samples were collected using the Quantisal oral fluid collection device (Immunalysis Corporation, Pomona, CA, USA).

To collect oral fluid, the collection pad was placed under the tongue or between the tongue and cheek for a maximum of 5 min, or until the volume indicator turned blue (indicating approximately 1 mL of collected oral fluid). Then, the pad was transferred to a collector tube containing 3.0 mL preservative buffer solution. The samples were stored at approximately 5 °C, until they were transported to the laboratory, and frozen at - 20 °C the following day. The samples were weighed to determine the volume of collected neat (undiluted) oral fluid using an XS802S balance (Mettler-Toledo AG, Greifensee, Switzerland) with a readability of 0.01 g. According to the manufacturer, the balance had a repeatability limit of 0.008 g and a linearity deviation limit of 0.020 g, although the typical values were reported as 0.004 g and 0.007 g, respectively.

Samples with a weight indicating that < 0.1 mL of neat oral fluid was collected were excluded from further analysis, as the inaccuracy of the balance would significantly affect results.

The samples were stored at - 20 °C before alcohol analysis was performed using an automated enzymatic method based on alcohol dehydrogenase with an AU680 Clinical Chemistry Analyser (Beckman Coulter, Brea, USA). Calibration and control were performed each day of sample analysis. The intermediate precision relative standard deviation (RSD) was 3.2% at an alcohol concentration of 0.2 g/L and 1.7% at 1.0 g/L. All analyses were performed by qualified laboratory personnel.

The alcohol concentration (C) in the neat oral fluid was calculated as follows:

C = C_Sample_ × (W_Buffer_ + (W_Sample_ - W_Empty_)) / (W_Sample_ - W_Empty_),

where C_Sample_ is the alcohol concentration in the collected oral fluid + buffer mixture, W_Buffer_ is the weight of the buffer in the oral fluid sampling device (3.0 g), W_Sample_ is the weight of the sampling device, including oral fluid sample, and W_Empty_ is the weight of the sampling device before use.

An alcohol concentration > 0.10 g/L was regarded as positive.

The laboratory is accredited by the National Accreditation Body for the analysis of alcohol and drugs in biological samples.

### Statistical analysis

Statistical analysis was performed using Microsoft Excel (Microsoft, Redmond, USA) and SPSS version 26 (IBM Corp., IBM Corporation, Armonk, USA).

For qualitative comparison, we calculated “true positives” defined as an alcohol concentration in neat oral fluid above the cutoff for an individual who had a breath alcohol concentration exceeding the cutoff; “false negatives” was defined as an alcohol concentration in neat oral fluid below the cutoff for an individual with a breath alcohol concentration exceeding the cutoff. “True-negative” and “false-positive” were defined similarly.

For quantitative comparison, we calculated the Intraclass Correlation Coefficient (ICC) using the two-way mixed model, including 95% confidence internal. An ICC of 0.75 or more was regarded as good, and 0.50 to 0.74 as moderate.

## Results

In the present study, we recruited 95 nightclub patrons. Oral fluid samples from six participants were excluded owing to low sample weights.

Among the remaining 89 participants, 88 tested positive for alcohol both in breath and in oral fluid; one participant tested negative in both matrices. Thus, no false-negative or false-positive results were obtained in oral fluid when compared with the breath.

**Figure 1:**
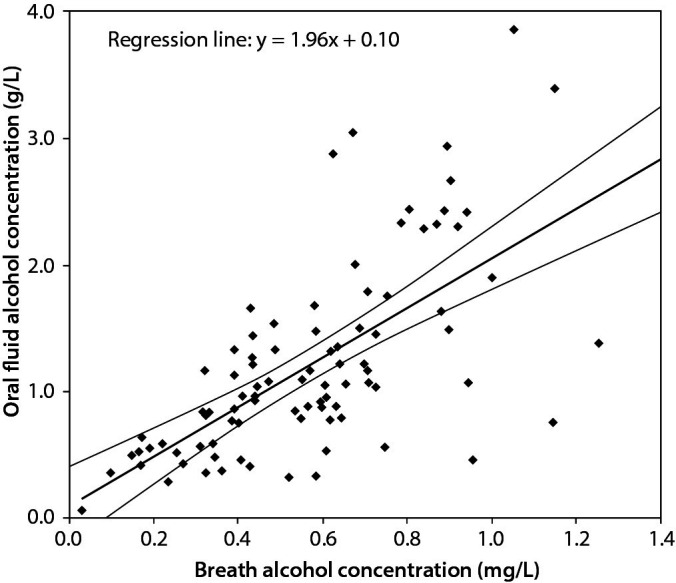
Quantitative comparison of alcohol concentrations in oral fluid and breath among 89 nightclub patrons.

Figure 1. presents alcohol concentrations in the oral fluid samples and breath alcohol concentrations. The ICC was 0.40 (95% CI 0.21-0.56; P > 0.001), which is poor.

## Discussion

We found a poor ICC between alcohol concentrations in the breath and oral fluid. Interestingly, a large proportion of the oral fluid samples showed lower alcohol concentrations than expected, and many had higher alcohol concentrations in oral fluid than breath testing would indicate. These findings could be due to deviation from the manufacturer’s recommendation for oral fluid collection, which should be performed at least 10 min after consuming food or beverage, and breath alcohol analysis should be performed at least 20 min after consuming food or beverage (*9*, *10*). However, the qualitative determination of alcohol in oral fluid matched the presence of alcohol in breath; drinking water did not reduce the alcohol concentration in oral fluid below the cutoff of 0.1 g/L in any case that tested positive for breath alcohol.

In a previous Polish study assessing 49 volunteers who were given controlled amounts of alcohol, followed by the sampling of oral fluid and breath at specified time points, a good correlation between estimated BAC and alcohol concentration in the oral fluid was documented; the difference in estimated BAC and concentration in the oral fluid was - 0.034 ± 0.080 g/L (*12*).

Jones has earlier performed a study with 10 volunteers who drank 0.80 g ethanol/kg body weight. He tested 124 oral fluid samples using the Q.E.D. device and compared with the breath alcohol concentration. He reported a good correlation between the two methods (*4*).

Previous studies have also found very good correlations between alcohol concentrations in oral fluid and blood. For example, McColl *et al.* compared alcohol concentrations in 300 paired oral fluid and blood samples in a study of 12 healthy males who ingested 100-200 mL alcohol (*2*). In another report by Jones, he compared alcohol concentrations in 168 paired oral fluid and blood samples from 21 healthy men who ingested ethanol at 0.68 g/kg body weight (*3*). Gubala and Zuba also reported a good correlation in a study evaluating 38 volunteers with 1152 paired alcohol concentrations in oral fluid and blood; the participants received 0.6-0.7 g/kg of body weight ethanol (*13*). However, most of the studies mentioned above made their conclusions based on visual assessment of scatter plots and calculation of the Pearson’s correlation coefficients, which requires that both samples follow a normal distribution. They neither calculated the ICC nor calculated bias; therefore, the reported correlations remain questionable.

Our findings demonstrate that the alcohol concentration in oral fluid samples collected shortly after drinking water did not accurately reflect the alcohol concentration in breath, as illustrated by the poor ICC.

This study has some limitations. The number of participants was small and not selected randomly; we did not record the time and type of last food and beverage consumed. We expect that factors other than the sampling procedure may also impact the accuracy and precision of the estimated alcohol concentrations but were of less importance; this may include variable buffer volume in the sampling device causing inaccurate weight (and volume) estimation of collected oral fluid, imprecision of the balance used to weigh samples, imprecision of the analytical methods, and concentration changes during storage of samples.

The procedure for the oral fluid collection was suitable for the qualitative determination of alcohol intake but not for quantitative estimation. Therefore, we recommend that oral fluid samples should not be used for estimating the blood or breath alcohol concentration among individuals who have recently consumed alcohol or non-alcoholic beverages, as stated in the instructions for use, unless the mouth is rinsed with water for at least 15 min before oral fluid is collected.
